# Incontinence after laparoscopic radical prostatectomy: a reverse systematic review

**DOI:** 10.1590/S1677-5538.IBJU.2021.0632

**Published:** 2022-01-28

**Authors:** Wilmar Azal, Diego M. Capibaribe, Luciana S. B. Dal Col, Danilo L. Andrade, Tomas B. C. Moretti, Leonardo O. Reis

**Affiliations:** 1 Universidade Estadual de Campinas Departamento de Urologia Campinas SP Brasil Urociência; Departamento de Urologia da Universidade Estadual de Campinas - UNICAMP, Campinas, SP, Brasil; 2 Pontifícia Universidade Católica de Campinas Campinas SP Brasil Pontifícia Universidade Católica de Campinas, PUC-Campinas - Campinas, SP, Brasil

**Keywords:** Prostatic Neoplasms, Prostatectomy, Urinary Incontinence, Systematic Reviews as Topic

## Abstract

**Purpose:**

To report the prevalence of the definitions used to identify post-prostatectomy incontinence (PPI) after laparoscopic radical prostatectomy (LRP), and to compare the rates of PPI over time under different criteria.

**Materials and Methods:**

In the period from January 1, 2000, until December 31, 2017, we used a recently described methodology to perform evidence acquisition called reverse systematic review (RSR). The continence definition and rates were evaluated and compared at 1, 3, 6, 12, and >18 months post-operative. Moreover, the RSR showed the “natural history” of PPI after LRP.

**Results:**

We identified 353 review articles in the systematized search, 137 studies about PPI were selected for data collection, and finally were included 203 reports (nr) with 51.436 patients. The most used criterion of continence was No pad (nr=121; 59.6%), the second one was Safety pad (nr=57; 28.1%). A statistically significant difference between continence criteria was identified only at >18 months (p=0.044). From 2013 until the end of our analysis, the Safety pad and Others became the most reported.

**Conclusion:**

RSR revealed the “natural history” of PPI after the LRP technique, and showed that through time the Safety pad concept was mainly used. However, paradoxically, we demonstrated that the two most utilized criteria, Safety pad and No pad, had similar PPI outcomes. Further effort should be made to standardize the PPI denomination to evaluate, compare and discuss the urinary post-operatory function.

## INTRODUCTION

The rate of prostate cancer (PCa) detection is currently increasing, and so is the rate of radical prostatectomy (RP). Urinary incontinence is a potential adverse event after RP for PCa, which leads to a significant worsening of quality of life (1).

Nevertheless, there is no international consensus on the optimal way to define, assess and grade post-prostatectomy incontinence (PPI). This heterogeneity in evaluating this aspect potentially explains the different prevalence rates reported, detains ideal comparison, and delays enlightenment of the question. The most common definitions of continence when evaluating PPI are based on the use of pads; however, they exclude patients who report any leakage - with no pad use-, which is the definition of incontinence promoted by the International Continence Society (ICS) (2, 3). Also, widely used criteria are the “no leak”, “no pad” or “safety pad”, while some are still grading PPI through a validated symptom scale of objective and subjective experience (4).

Our primary objective was to report the prevalence of the definitions used to identify PPI after laparoscopic radical prostatectomy (LRP), and secondly, to compare the rates of PPI overtime under these different criteria. To reach this propose we used a novel methodology described by our group, which can delineate the “natural history” of an issue reported along its timeline, called reverse systematic review (RSR). In the RSR, clinical evidence starts from general information, in this case, LRP, to then is filtered until it reaches a specific knowledge, such as PPI, through a wide search criterion (5).

## MATERIALS AND METHODS

Since reverse systematic review (RSR) is a novel methodology, it is not possible to register in international database of prospectively registered systematic reviews such as PROSPERO.

Systematized research for evidence acquisition for the RSR was carried out in January 2018 and we searched systematic reviews (SR) articles, with or without meta-analysis, that approached the topic LRP. We did not study papers in 2019 and 2020 because there were very few SR about LRP in this period, since robotic assisted laparoscopic prostatectomy predominates more recently. The databases used were: PubMed, Web of Science, Cochrane Library, Embase, ProQuest, CINAHL (The Cumulative Index to Nursing and Allied Health Literature), BVS/Bireme, and Scopus. Only papers in English were considered for our search, in a period within January 1, 2000, until December 31, 2017. Reviews without a clear and systematized search methodology, integrative methodology, expert consensus, and abstracts or summaries were excluded.

After we identified the two most used criteria of continence when assessing PPI (No pad and Safety pad), results were divided into three groups: No pad, Safety pad, and Others (No leak, any score, no precise information). The continence rates were evaluated at 1, 3, 6, 12, and >18 months post-operative. Finally, we compared the respective criteria of continence reported and their rates at the different post-operative moments.

Descriptive and arithmetic methods were used to describe the samples (mean and median) and dispersion (standard deviation, standard error of the mean, and confidence interval). Parametric distributions were compared through one-way ANOVA and post-hoc analysis with Bonferroni’s correction. Otherwise, non-parametric distributions were compared with the Kruskal-Wallis test. In all analyses, a significance level of 5% (p <0.05) was used for 2-tailed interpretation. Calculations were performed with the IBM SPSS Statistics v.24.

## RESULTS

**Figure 1:**
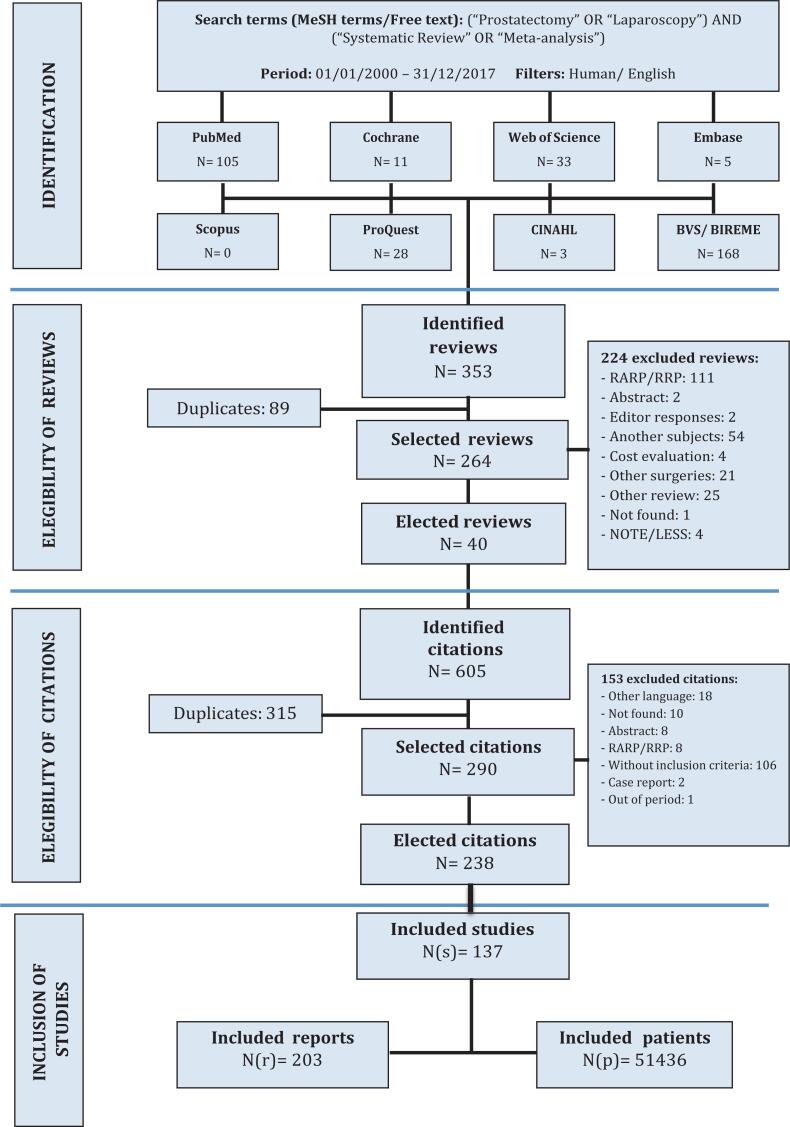
This figure shows the design of the search strategy by a flowchart, which illustrates the eligibility process of systematic reviews and primary studies to compose the final database.

We identified 353 review articles in the systematized search. After the exclusion of duplications and filters by the inclusion and exclusion criteria, 40 reviews were chosen, which cited 605 articles about LRP. After exclusion of doubling and eligibility criteria, 137 studies about PPI were selected for data collection, finally including 203 reports (nr) and 51.436 patients (Figure-1).

In all the post-operative timeframes, the most used criterion of continence was No pad (nr=121; 59.6%), the second one was Safety pad (nr=57; 28.1%), followed then by a small number of other classifications (nr=25; 12.3%). Accordingly, results were divided into three groups: No pad, Safety pad, and Others.

**Figure 2:**
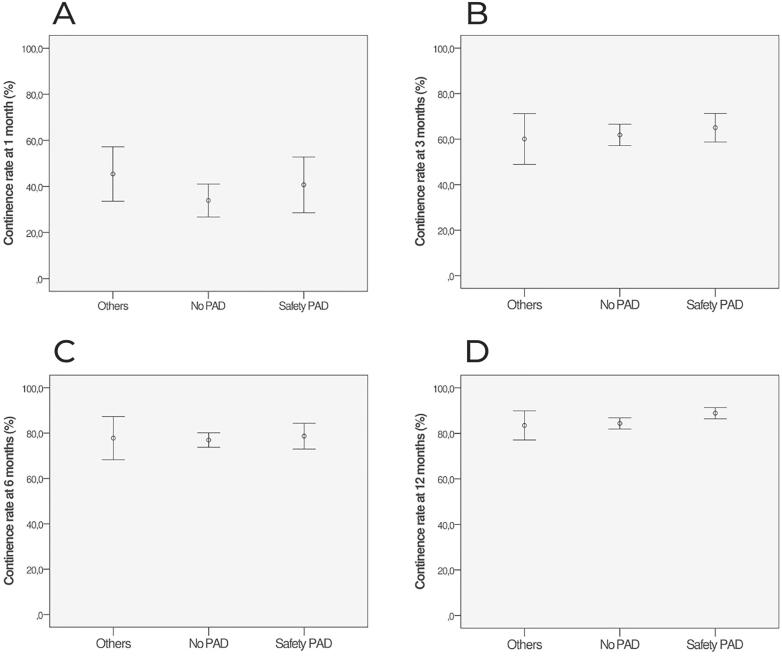
A-D The figures show the continence rate (% mean and SE with 95% CI) after LRP over time, at 1 (A), 3 (B), 6 (C) and 12 (D) months, stratified by three continence criteria (No PAD; Safety PAD; Others).

**Figure 3:**
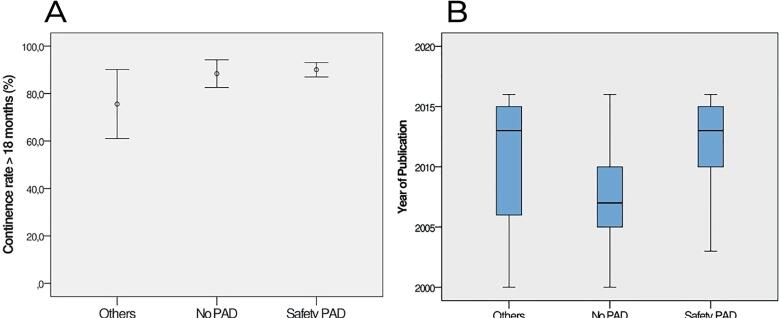
A-B The figure A shows the continence rate (% mean and SE with 95% CI) after LRP at >18 months. The figure B shows the boxplot distribution of year of publication of the studies, stratified by three continence criteria (No PAD; Safety PAD; Others) with median comparisons by Kruskal-Wallis test (p<0.001).

The PPI after LRP was evaluated in each of these three continence criteria in each specific post-operative period. A statistically significant difference between criteria was identified only at >18 months (p=0.044). The post-hoc analysis at this timeframe showed that there was no difference between No pad vs. Safety pad (p=0.699), but there was statistical significance between No pad vs. Others (p=0.023) and Safety pad vs Others (p=0.015), Table-1, Figures 2A-D and 3A.

**Table 1:** Continence rate over time in each criterion.

Continence criteria	1 month		3 months		6 months		12 months		> 18 months	
	n_R_	%Mean, SE	n_R_	%Mean, SE	n_R_	%Mean, SE	n_R_	%Mean, SE	n_R_	%Mean, SE
**No PAD**	34	33.9 (3.6)	75	61.8 (2.4)	75	76.9 (1.6)	95	84.4 (1.2)	19	88.4 (2.9)
**Safety PAD**	16	40.7 (6.1)	36	65.1 (3.1)	21	78.6 (3.8)	32	88.8 (1.2)	14	90.0 (1.5)
**Others**	3	47.2 (12.8)	7	69.6 (11.7)	3	84.8 (10.5)	5	81.1 (4.9)	4	72.5 (11.7)*
**Total**	53	36.7 (3.0)	118	63.3 (1.9)	99	77.6 (1.4)	132	85.3 (0.9)	37	87.3 (2.1)
**p**	0.418		0.529		0.570		0.089		0.044*	

n_R_ = number of reports; SE = standart error of the mean

* p < 0.05

Regarding the evaluation of the continence criteria used along the manuscript year of publication, there is a significant difference among No pad (median=2007) and the other two criteria, Safety pad (median=2013) and Others (median=2013), with p <0.001. It was observed that until 2007 the No pad criteria predominated. From 2013 until the end of our analysis, the Safety pad and Others were the most reported, Figure-3B.

## DISCUSSION

Urinary incontinence is still today one of the main concerns after RP for PCa treatment, despite the improvement in technique and technology, afflicting up to 74% of patients undergoing this surgery in the short term (3, 6). Various factors have been hypothesized to contribute to this undesired outcome. Potential preoperative variables might be age, body mass index, prostate size, oncological factors, preoperative urinary and sexual dysfunction, prior transurethral resection of the prostate, membranous urethral length before surgery (7-9).

In addition to that, hypothetic intraoperative factors that could influence PPI include puboprostatic ligament sparing, surgical neuro-vascular bundle approach, preservation of the endopelvic fascia, selective suturing of dorsal venous complex, size of the urethral stump, bladder neck preservation, posterior or anterior reconstruction supports, and others (7, 8, 10, 11).

Moreover, cultural differences in the way the patient reports urinary leakage and pad use could also interfere in the analyzed outcomes (12). PPI has been the topic of studies ever since the first patient came back for a follow-up after surgery and urologists have been comparing notes and studies to find a better way of avoiding this complication. Urinary continence is known to be a strong determinant of quality of life; however, how much incontinence is “too much”? How much incontinence is incontinence and how to compare this outcome? Studies show very heterogenous PPI results, ranging from 3 to 74% (5, 6, 11).

Surgeons may operate the same surgery, with the same intent, similar cases, but they are different and might have different results. It is imperative to find a pattern to graduate PPI, to compare the outcomes obtained in clinical practice. Loughlin and Prasad described methodological instruments and definitions used for PPI: pads use/number, pad weight, urodynamics assessment, validated questionnaire, institutional questionnaire, phone or face-to-face interview (8). Still, there is a need to homogenize the approach to the same concept or question to be able to evolve in the right direction. This study tries to demonstrate the prevalence of the definitions used to identify PPI after LRP as time goes by and to compare the PPI rates overtime under these different terminologies.

We used a new methodology to perform our evidence acquisition: reverse systematic review (RSR). Conceptually, a standard systematic review (SR) allows the selection of eligible primary studies to answer a specific clinical question, and this method inherently eliminates multiple secondary variables. In the RSR, clinical evidence starts from general information, in this case, LRP, to then be filtered until it reaches a specific knowledge, such as PPI. It follows the opposite path of an SR, going from the specific data of the systematic revisions and then back to its primary studies. Also, the RSR has a wide search criterion, heterogeneous eligibility, and an intention of studying the development through temporal correlation (5).

We found the main criteria used for classifying PPI after LRP: No pad and Safety pad. Yet, most publications have been using “pads” to measure the degree of incontinence, and although trying to be quantitative, they are still subjective (13). Moreover, as referred above, some investigators proposed to weigh the pads, since some patients only use them for safety (14). Scores are acceptable to evaluate continence, as seen in many cases (3, 5), but validation, translation, comprehension by patients and doctors sometimes are yet challenging barriers to overcome.

Many authors studied this topic and even tried to propose a new standardization. Ellison et al. in 2013 (4) propounded a stratification of the Expanded Prostate Cancer Index Composite - Short Form Urinary Domain (EPIC-UIN) (which contains 3 questions related to urinary function and 2 to urinary bother) to simplify the results in a “meaningful fashion”: mild, moderate and severe incontinence. These levels are already used by another classification, the Incontinence Severity Index (ISI) (which contains 3 questions about urinary stress incontinence, 3 about urinary urge incontinence, and 2 related to pad number and type). Even though they stated that this system would aid physicians and doctors to interpret the PPI status, as they found an agreement of 74.1% when comparing both instruments, when observing publications afterward, it is seen that this stratification did not become a standard.

In this manner, Holm et al. in a prospective study, concluded that PPI varied considerably according to the definition applied, and any leakage is incontinence. The authors also stated that “further effort should be made to reach consensus on PPI severity grading” (3). Considering the finding related to the two most used criteria, one should think that the simpler the PPI described, the better it is. However, both continence criteria came with their theories for limitations. Could a more restrictive classification such as No pad diminish continence ratios to a grimmer scenario? Could the Safety pad concept for characterizing a patient as a continent lower the bar enough to rate as continent people that are suffering from significant leakage? What is the real difference between these two parameters?

The RSR responded to the last one. It proved to be useful in demonstrating the “natural history” of the evolution of PPI after LRP, which is not captured by a standard SR. Its reverse methodology was able to demonstrate the stability of the population sample obtained even in different scenarios, allowing a comparison of the main criteria used for this purpose along the timeline. This stability, from a statistical point of view, is proven by the substantial sample size and reduced standard error of the mean, since incorporating any additional value in the analysis would hardly change the overall mean.

Interestingly, this study reported that not only there are no differences in continence ratios in the two main criteria at 1, 3, 6, and 12 months after surgery, but also there is no difference when comparing other denominations used for PPI in some publications (which we labeled “Others”). The mean continence rates at these periods, respectively, were: 36.7%, 63.3%, 77.6%, and 85.3%. Statistically, a significant value was only seen at 18 months post-operative (p=0.044). However, at this time frame we have the fewest total number of studies (nr=37), mainly due to the minimum clinical improvement after 12 months of RP (15), -and our results corroborate with this data, with an improvement rate of 2.0% when comparing 12 vs. 18 months post-operative (85.3±0.9 vs. 87.3±2.1%). Moreover, further analysis at this latest period showed no difference between No pad vs. Safety pad (p=0.699) and only observed statistical significance between No pad vs. Others (p=0.023) and Safety pad vs. Others (p=0.015).

The fact that there was no significant difference in the result’s rates in any post-operative period between both PPI main denominations may indicate that they measure the same thing in a very similar way. The subtle difference between groups may point to a need to investigate other parameters affecting the quality of life that may differ between these patients. The Safety pad offers real benefits, or is it just like safety wheels after you have already learned how to ride a bike? Eventually, a better conversation with the patient as to the usefulness of this “Security pad” might end in a more incisive recommendation by the urologist to just do not use it at all.

Time frame analysis of publications (Figure-3B) documented the already mentioned “natural history” of the use of these PPI criteria, which is another peculiar aspect of the RSR. It depictured that most authors preferred to use the Safety pad terminology after 2011, probably in pursuit of better continence outcomes. Likely, many other authors then followed the same path from that point on, inferring in a herd mentality, which resulted in a wave swooping after previous publications in which prevailed other criteria. Very interestingly, we verified that this attempt to raise continence percentages by allowing the Safety pad parameter to enter the “continent group” did not impact continence rates in most scenarios, and only lead to a false sensation of better outcomes in PPI.

This study is not free of limitations. The composition of a heterogeneous sample allows the temporal analysis but prevents the defragmentation of variables to analyze specific outcomes, a characteristic of SRs with meta-analysis. The presence of correlations with reduced coefficients of determination limits the statistical strength despite being a common feature in heterogeneous population samples. Further multivariate analysis, such as stratification by other variables that may influence continence after surgery, may reduce such interference.

In addition, even if the isolated samples show asymmetric distributions, they are small enough to contribute a significant weight alone, and the overall size is high enough to minimize or even avoid such bias. Finally, the results found in this study must be evaluated with caution regarding biases when comparing continence rates after LRP before and after 2011, especially if one is inclined to compare them to open radical prostatectomy and robotic-assisted radical prostatectomy (RALP) rates. This novel methodology of RSR also allows for PPI measurement criteria and their rates to be compared in future studies, including RALP, which is the main RP technique currently (16-18). Its strength is beyond scientometrics, adding to the evidence-based medicine by temporally analyzing the surgical complication in question, allowing possible projections for the future, and exposing significant bias neglected by SRs.

## CONCLUSION

Reverse Systematic Review revealed the “natural history” of PPI after the LRP technique. Beyond that, the study analyzed the criteria used to evaluate PPI and showed that through time, with the presumable objective of reaching better continence results, the Safety pad concept was mainly used. However, paradoxically, we demonstrated that the two main criteria utilized, Safety pad and No pad, had similar PPI outcomes. Further effort should be accomplished to reach an international consensus on a clear and objective concept for PPI so that a more accurate comparison between studies and techniques of RP can be achieved.
